# Vascular plants occurrences in the Southern Urals industrial towns (Sterlitamak and Salavat)

**DOI:** 10.3897/BDJ.10.e77148

**Published:** 2022-03-02

**Authors:** Yaroslav Golovanov, Larisa Abramova, Mikhail Drap, Maria Lebedeva

**Affiliations:** 1 South-Ural Botanical garden-institute, Ufa Federal Scientific Centre, Russian Academy of science, Ufa, Russia South-Ural Botanical garden-institute, Ufa Federal Scientific Centre, Russian Academy of science Ufa Russia

**Keywords:** biodiversity, urban flora, plant species, vascular plants, alien species, Salavat, Sterlitamak, the Southern Urals, Republic of Bashkortostan

## Abstract

**Background:**

The paper presents datasets of plant species of two industrial cities Sterlitamak and Salavat (Republic of Bashkortostan) is presented. These cities are part of the Southern Bashkortostan urban agglomeration and are amongst the three largest in the Republic. The population of Sterlitamak is about 276,000. There are large oil refineries and other large industrial transport infrastructure facilities. Datasets are prepared on the basis of long-time field research by Ya. Golovanov (2008 - 2016). Technical preparation of the datasets was carried out by M. Lebedeva and M. Drap. The herbarium samples are stored in the herbarium collections of the South Ural Botanical Garden Institute and the Ufa Institute of Biology (UFA). The data paper describes three datasets on species occurrences. It presents occurrences of species in different types of habitats (anthropogenically transformed and semi-natural). The datasets consists of 5,462 occurrence records totally. Most of the records (5,359) are georeferenced.

**New information:**

The total number of records in three datasets is 5,462. They contain of vascular plant species occurrences in the two industrial cities of the Southern Urals (Sterlitamak and Salavat). There are both alien and natural species occurrences in different types of habitats (antropogenically transformed and semi-natural).

## Introduction

The rate of urbanisation on a global scale is rapidly increasing. Today, about 50% of the world's population lives in cities; in Europe, this proportion is 70% (United Nations 2008). Urbanisation is a major anthropogenic effect that can lead to biotic homogenisation (Lososová et al. 2012, Trentanovi et al. 2013, Aronson et al. 2014). An increase in the anthropogenic impact on ecosystems currently leads to synanthropisation, replacing the species of natural communities with the synanthropic and alien species resistant to anthropogenic effects. Replacing natural plant communities with the synanthropic ones, reducing biodiversity, simplifying the structure, reducing the productivity and stability of plant communities are taking place (Abramova and Mirkin 2000). However, urban areas belong to the types of landscapes with a high species richness. Often, more species are represented on the territory of the cities than on the adjacent territories. The main feature of urban flora is a high proportion of alien plant species (von der Lippe and Kowarik 2008). This phenomenon has been observed in various regions of the world - Europe, North America, South America (for example, Balmford et al. 2001, Araujo 2003). At the same time, forest, steppe, meadow, meadow-steppe elements of the flora, which reflect the zonality of the vegetation cover, are preserved in highly fragmented natural habitats in urban landscapes. These territories also make a significant contribution to the floristic species richness of urban ecosystems.

The study of the urban flora has been carried out in the Republic of Bashkortostan over the years by a number of authors (e.g. Ishbirdina and Ishbirdin 1993, Ryabova 1996, Edrenkina, V. À. 2005, Golovanov and Abramova 2016, Golovanov and Abramova 2017, Golovanov et al. 2017).

In this paper, we consider two industrial cities, Salavat and Sterlitamak, which are part of the Southern Bashkortostan urban agglomeration and are located on the border of the steppe and forest-steppe zones. In the past, almost all treeless flat spaces in this area were covered by herbaceous-bunchgrass steppes, dominated by *Stipacapillata* L., with an admixture of *Stipalessengiana*, *S.pennata*, *S. korshinskyi, Festuca pseudovina* and various meadow steppe herbal species. On the edges of forests, in wetter habitats and on the northern slopes, meadow steppes developed with the presence of feather grass and an abundance of legumes. On shallow chernozems, there were bunch-grass steppes, almost devoid of herbs. Saltwater meadows developed in the floodplains of the rivers (Kadilnikov 1964).

Due to the long history of economic development, as well as the high pace of construction, the preserved areas of natural vegetation (steppe and meadow habitats) within the city boundaries were mainly destroyed. At the moment, the main types of vegetation cover of the city are plots with various kinds of ruderal (secondary) habitats. In the floodplains of the rivers, there are ruderalised meadows, areas of insulated and wet meadows, steppes, floodplain forests, as well as a complex of aquatic and coastal-aquatic vegetation.

The variety and main patterns of vegetation differentiation in Sterlitamak and Salavat are considered in the works of Ya. Golovanov (Golovanov and Abramova 2017, Golovanov et al. 2017, Golovanov 2018).

Prepared according to the concept of "data paper" (Chavan and Penev 2011), this paper aims to present datasets of vascular plants occurrences of the Southern Urals industrial cities (Sterlitamak and Salavat), which are published in GBIF as a Darwin Core Archive.

## Project description

### Title

Vascular plants occurences of the Southern Urals industrial cities (Sterlitamak and Salavat)

### Personnel

Yaroslav Golovanov, Larisa Abramova, Mikhail Drap, Maria Lebedeva

### Study area description

Salavat and Sterlitamak are important industrial cities in the Republic of Bashkortostan (Russia). The population of Salavat is more than 159,000 people, Sterlitamak - 279,000. These towns are included in the Southern Bashkortostan urban agglomeration and are located in the Southern Urals on the border of the steppe and forest-steppe zones. The Urals are a mountain range that runs almost continuously along the 60°E meridian from the Arctic Ocean coast to the Ural River and north-western Kazakhstan. The Urals are an important botanical and geographical boundary of Northern Eurasia.

### Design description

The study of the flora and vegetation diversity of Salavat and Sterlitamak was carried out from 1999 to 2019 by Ya. Golovanov using route observation and sample plots. The main results and analytics were published in number of papers (Golovanov and Abramova 2017, Golovanov et al. 2017, Golovanov 2018). The datasets were prepared according to the data of field research (releves and species lists). Some species are deposited in the herbarium of South-Ural Botanical Garden Institute and Institute of Biology, Ufa Scientific Center of the Russian Academy of Sciences (UFA). These datasets include information on the occurrence of 292 species from 185 genera and 48 families. The total number of occurences is 5,462.

### Funding

This work was supported in project no. АААА-А18-118011990151-7 of the Ufa Federal Scientific Centre of the Russian Academy of Sciences.

## Sampling methods

### Study extent

The datasets include 5,462 occurrences of vascular plant species in Sterlitamak and Salavat, industrial cities of the Republic of Bashkortostan. The study of the flora and vegetation of Sterlitamak was carried out in 2014-2015 (May-August). During this period, 360 releves of the main types of anthropogenic and natural vegetation were made within the city boundaries. The main studies of the flora and vegetation of Salavat were carried out from 2008 to 2011 (May-August). Some types of communities were described later in 2016. During this period, 500 releves of anthropogenic and natural types of vegetation were made. The regions are located in the Southern Urals on the border of steppe and forest-steppe vegetation zones. The datasets contain information on 292 species, 185 genera, 48 families and two classes (Liliopsida and Magnoliopsida).

### Sampling description

Comprehensive studies of the flora and vegetation of cities were carried out in anthropogenic and natural habitats. Research routes covered all areas of the cities, as well as adjacent territories. Typical anthropogenic habitats include: railway stations, slopes of roads and railways, wastelands, construction sites, abandoned gardens, anthropogenic reservoirs, city yards and lawns. Such habitats are characterised by a high concentration of alien plant species. In these territories, releves of the main anthropogenic types of plant communities were carried out. The locations of individual alien plant species were recorded outside the sample plots. Routes were also mapped in the areas of natural vegetation: meadows, steppes, forests and their edges and banks of water reservoirs. In these territories, releves of plant communities were carried out, rare species of plants were recorded additionally. Releves were carried out on sample plots of various sizes (4-100 m^2^) depending on the boundaries of phytocoenoses in accordance with the methods of ecological-floristic classification of Braun-Blanquet. The scientific names of plants were adjusted in accordance with the International Plants Names Index (http://www.ipni.org).

### Quality control

The identification of plants was carried out according to "Flora of the European part of the USSR" (1974-1994) and "Flora of Eastern Europe" (1996-2004). All releves are included in the database of anthropogenic vegetation of the Urals and adjacent territories (http://www.givd.info/ID/00-RU-008), implemented using the TURBOVEG software (Hennekens and Schaminée 2001). Herbarium materials are stored in the South-Ural Botanical Garden Institute, Ufa Federal Scientific Centre, Russian Academy of Science and Ufa Institute of Biology, Ufa Federal Scientific Centre, Russian Academy of Science (UFA).

### Step description

The main habitats and types of anthropogenic, semi-natural and natural plant communities in cities are identified. For each type of vegetation, at least 10 geobotanical releves are made on 10 m * 10 m plots or within the boundaries of phytocoenoses. Additionally, spots of the alien species sightings outside the plots were recorded. The releves are compiled in the Turboveg electronic database and are presented in the semi-restricted access mode in the European vegetation archive (EVA). Then, species occurrences data are collected as datasets. The dataset fields’ names were chosen according to Darwin Core (Wieczorek et al. 2012) and include the following: «occurrenceID», «basisOfRecord», «kingdom», «phyllum», «class», «order», «family», «genus», «scientificName», «taxonRank», «countryCode», «recordedBy», «eventDate», «day», «month», «year», «identifiedBy», «decimalLatitude», «decimalLongitude», «stateProvince».

## Geographic coverage

### Description

The geographic coverage of the study included two cities, Serlitamak and Salavat, which are located in the Southern Urals on the border of the steppe and forest-steppe zones.

Sterlitamak - a city in the Republic of Bashkortostan, the administrative centre of the Sterlitamak District (since 1930), is located 121 km south of Ufa. Sterlitamak is located in the floodplain of the Belaya River, as well as its medium and small tributaries - the Ashkadar, Sterlya, Seleuk and Olkhovka Rivers. The population is 279 thousand people (as of 2016). The area of the city is 108.52 km², the population density is 2577.33 people/km². The city is a major junction of the Ufa-Orenburg railway and republican and federal highways and one of the centres of the South Bashkortostan polycentric agglomeration. This territory has a powerful production potential with a total population of about 600 thousand people. Sterlitamak was founded in 1766 as the Ashkadar salt marina on the Sterlya River and was, at that time, in the territory of the Orenburg Province. In 1919-1922, Sterlitamak was the capital of the Autonomous Bashkir Soviet Republic, from 1920 - the centre of the Sterlitamak canton. With the discovery of oil near Ishimbay, the city became one of the strongholds for the development of oil fields. Now, Sterlitamak is a large centre of the chemical industry. In the territory of the city, there are large chemistry enterprises, as well as enterprises in the machine building industry.

Salavat was founded on 30 June 1948 as the village Novostroyka due to the construction of the petrochemical plant. In 1949, Salavat received the status of an industrial township and, in 1954, the status of a city. Currently, Gazprom Neftekhim Salavat is a major centre for the oil refining and petrochemical industries. In addition, there is a large glass factory (Salavatsteklo), as well as a plant for reinforced concrete and mineral wool products (Zykina 1998). Salavat is the third largest city of the Republic of Bashkortostan. The population is 156 thousand people. The length of the residential territory of the City along the Belaya River is 5.5 km, in width - 2.7 km. In addition to the residential part, the large territory of the City is occupied by industrial and economic buildings. The area of the City together with industrial territories is 111.4 km². Communication routes significant for the Republic of Bashkortostan pass through the City of Salavat: the Ufa-Orenburg highway and the Salavat railway.

The main climatic characteristics of the cities are the following. The average annual air temperature here is 3.2°C, the average January temperature is -13.9°C, July is + 19.4°C, the sum of positive temperatures above 10°C is 2376°C. The average annual rainfall is 498.9 mm (Salavat) and 576 mm (Sterlitamak). The average precipitation equals 285 mm and 169 mm during the growing season and during the winter, respectively, snow cover height reaches 41 cm, soil freezing depth is 61 cm. The final spring frosts are observed in the second or third decade of May, the first autumn frost occur in the first or second decade of September. The frost-free period averages 128 days (Taichinov and Bulchuk 1975, Yaparov 2005).

### Coordinates

53.361651 and 53.630403 Latitude; 55.924681 and 55.930825 Longitude.

## Taxonomic coverage

### Description

The datasets include records on natural and alien species belonging to Magnoliophyta, 48 families, 185 genera and 292 species. The largest number of species (197) belong to the families Asteraceae, Poaceae, Amaranthaceae, Brassicaceae, Fabaceae, Apiaceae, Rosaceae, Lamiaceae and Polygonaceae. Additionally, Asteraceae (1705), Poaceae (641), Amaranthaceae (522), Brassicaceae (474) and Fabaceae (361) account for the highest number of occurrences. Twenty families are characterised by just a a few occurrences (less than 20). The families Alismataceae, Amaryllidaceae, Caprifoliaceae, Hypericaceae, Polemoniaceae and Rhamnaceae were represented by only one species and one occurrence (Table 1).

## Temporal coverage

### Notes

2008-2016

## Usage licence

### Usage licence

Creative Commons Public Domain Waiver (CC-Zero)

## Data resources

### Data package title

Vascular plants occurrences of the Southern Urals industrial towns (Sterlitamak and Salavat).

### Resource link

https://www.gbif.org/dataset/ef233925-3e68-43b6-ae69-9c5ec29b2630, https://www.gbif.org/dataset/bc265af7-09e9-4887-acbb-823bd59052b7, https://www.gbif.org/dataset/d6a5db54-ffb5-4399-bdfd-876d888d7184

### Alternative identifiers

https://doi.org/10.15468/sghwhv, https://doi.org/10.15468/hys54z, https://doi.org/10.15468/heqqwj

### Number of data sets

3

### Data set 1.

#### Data set name

alien_plants_of_sterlitamak

#### Data format

Darwin Core

#### Number of columns

20

#### Character set

utf8

#### Download URL

https://www.gbif.org/dataset/ef233925-3e68-43b6-ae69-9c5ec29b2630

#### Description

The dataset, containing the data on the alien species of Sterlitamak (Republic of Bashkortostan), is presented. The dataset includes one table of species occurrences and contains 1486 records of species, registered during geobotanical surveys (releves). It presents occurrences of alien species in different types of habitats (antropogenically transformed and semi-natural).

**Data set 1.** 

Column label	Column description
occurrenceID	An identifier for the Occurrence (as opposed to a particular digital record of the occurrence).
basisOfRecord	HUMAN_OBSERVATION
recordedBy	Yaroslav Golovanov
eventDate	The date-time or interval during which an Event occurred. https://dwc.tdwg.org/list/#dwc_eventDate
year	2013-2015
month	June-August
day	The integer day of the month on which the Event occurred. https://dwc.tdwg.org/list/#dwc_day
countryCode	RU
stateProvince	Bashkortostan
decimalLatitude	53.589544-53.725891
decimalLongitude	55.89311-56.043895
identifiedBy	Yaroslav Golovanov
scientificName	The full scientific name, with the authorship and the date information, if known. When forming part of an Identification, the name should be the lowest level taxonomic rank that can be determined. This term should not contain identification qualifications, which should instead be supplied in the Identification Qualifier term.
kingdom	Plantae
phylum	Tracheophyta
class	Liliopsida, Magnoliopsida
order	Alismatales, Apiales, Asterales, Boraginales, Brassicales, Caryophyllales, Ericales, Fabales, Gentianales, Lamiales, Malpighiales, Malvales, Myrtales, Poales, Ranunculales, Rosales, Santalales, Sapindales, Solanales, Vitales
family	42 families
genus	134 genus
taxonRank	SPECIES

### Data set 2.

#### Data set name

Alien plants of Salavat

#### Data format

Darwin Core

#### Number of columns

20

#### Character set

utf8

#### Download URL

https://www.gbif.org/dataset/d3b36936-d030-4ebb-a0f1-d046dcd947e4

#### Description

The dataset, containing the data of alien species of Salavat (Republic of Bashkortostan), is presented. The dataset includes one table of species occurrences and contains 874 records, registered during geobotanical surveys (releves). It presents occurrences of alien species in different types of habitats (antropogenically transformed and semi-natural). There are both woody and herbaceous species in the dataset.

**Data set 2.** 

Column label	Column description
occurrenceID	An identifier for the Occurrence (as opposed to a particular digital record of the occurrence).
basisOfRecord	HUMAN_OBSERVATION
recordedBy	Yaroslav Golovanov
eventDate	The date-time or interval during which an Event occurred. https://dwc.tdwg.org/list/#dwc_eventDate
year	2008-2016
month	June-September
day	The integer day of the month on which the Event occurred. https://dwc.tdwg.org/list/#dwc_day
countryCode	RU
stateProvince	Bashkortostan
decimalLatitude	53.32653-53.40157
decimalLongitude	55.88417-55.9024
identifiedBy	Yaroslav Golovanov
scientificName	The full scientific name, with authorship and date information, if known. When forming part of an Identification, this should be the name in the lowest level taxonomic rank that can be determined. This term should not contain identification qualifications, which should instead be supplied in the IdentificationQualifier term.
kingdom	Plantae
phylum	Tracheophyta
class	Liliopsida, Magnoliopsida
order	Alismatales, Apiales, Asterales, Boraginales, Brassicales, Caryophyllales, Cucurbitales, Ericales, Fabales, Lamiales, Malpighiales, Malvales, Poales, Ranunculales, Rosales, Sapindales, Solanales, Vitales
family	25 families
genus	60 genus
taxonRank	SPECIES

### Data set 3.

#### Data set name

Natural plant species of Sterlitamak

#### Data format

Darwin Core

#### Number of columns

20

#### Character set

utf8

#### Download URL

https://www.gbif.org/dataset/d6a5db54-ffb5-4399-bdfd-876d888d7184

#### Description

The dataset, containing the data on the natural plant species of Sterlitamak (Republic of Bashkortostan), is presented. It includes one table of species occurrences and contains 3102 records of species, registered during geobotanical surveys (releves). It presents occurrences of species in different types of habitats (antropogenically transformed and semi-natural).

**Data set 3.** 

Column label	Column description
occurrenceID	An identifier for the Occurrence (as opposed to a particular digital record of the occurrence).
basisOfRecord	HUMAN_OBSERVATION
recordedBy	Yaroslav Golovanov
eventDate	The date-time or interval during which an Event occurred. https://dwc.tdwg.org/list/#dwc_eventDate
year	2013-2015
month	June-August
day	The integer day of the month on which the Event occurred. https://dwc.tdwg.org/list/#dwc_day
countryCode	RU
stateProvince	Bashkortostan
decimalLatitude	53.589544-53.725891
decimalLongitude	55.89311-56.043895
identifiedBy	Yaroslav Golovanov
scientificName	The full scientific name, with authorship and date information, if known. When forming part of an Identification, this should be the name in the lowest level taxonomic rank that can be determined. This term should not contain identification qualifications, which should instead be supplied in the IdentificationQualifier term
kingdom	Plantae
phylum	Tracheophyta
class	Liliopsida, Magnoliopsida
order	Alismatales, Apiales, Asterales, Boraginales, Brassicales, Caryophyllales, Ericales, Fabales, Gentianales, Lamiales, Malpighiales, Malvales, Myrtales, Poales, Ranunculales, Rosales, Santalales, Sapindales, Solanales, Vitales
family	42 families
genus	134 genus
taxonRank	SPECIES

## Additional information

Lebedeva M, Yamalov S, Drap M, Abramova L, Golovanov Y (2021). Alien_plants_of_Sterlitamak. South-Ural Botanical Garden Institute of Ufa Federal Scientific Centre of Russian Academy of Sciences. Occurrence dataset https://doi.org/10.15468/sghwhv (Lebedeva et al. 2021a) 

Lebedeva M, Yamalov S, Drap M, Abramova L, Golovanov Y (2021). Alien plants of Salavat. South-Ural Botanical Garden Institute of Ufa Federal Scientific Centre of Russian Academy of Sciences. Occurrence dataset https://doi.org/10.15468/hys54z (Lebedeva et al. 2021c)

Lebedeva M, Yamalov S, Drap M, Abramova L, Golovanov Y (2021). Natural plant species of Sterlitamak. South-Ural Botanical Garden Institute of Ufa Federal Scientific Centre of Russian Academy of Sciences. Occurrence dataset https://doi.org/10.15468/heqqwj (Lebedeva et al. 2021b).

## Figures and Tables

**Table 1.** Taxonomic distribution of the species and the species occurrences amongst the families in the datasets. The families are listed in descending order according to the total species number included in the datasets.

**Family**	**Number of species**	**Number of genera**	**Records**
Asteraceae	51	32	1705
Poaceae	35	22	641
Brassicaceae	22	16	474
Fabaceae	19	8	361
Amaranthaceae	17	6	522
Lamiaceae	17	13	212
Apiaceae	13	13	120
Rosaceae	13	8	91
Polygonaceae	10	4	242
Caryophyllaceae	9	5	56
Plantaginaceae	7	4	144
Cyperaceae	7	3	8
Boraginaceae	6	6	158
Rubiaceae	6	1	33
Solanaceae	6	3	29
Convolvulaceae	5	3	195
Malvaceae	3	3	39
Salicaceae	3	2	10
Oleaceae	3	1	10
Scrophulariaceae	3	2	9
Sapindaceae	2	1	89
Euphorbiaceae	2	1	63
Papaveraceae	2	2	36
Ranunculaceae	2	2	28
Cannabaceae	2	2	24
Ulmaceae	2	1	13
Violaceae	2	1	11
Geraniaceae	2	2	6
Onagraceae	2	1	6
Primulaceae	2	1	4
Juncaceae	2	1	3
Urticaceae	1	1	60
Cucurbitaceae	1	1	15
Portulacaceae	1	1	12
Balsaminaceae	1	1	6
Asparagaceae	1	1	4
Lythraceae	1	1	5
Typhaceae	1	1	5
Thesiaceae	1	1	4
Hydrocharitaceae	1	1	2
Vitaceae	1	1	2
Alismataceae	1	1	1
Amaryllidaceae	1	1	1
Caprifoliaceae	1	1	1
Hypericaceae	1	1	1
Rhamnaceae	1	1	1
Adoxaceae	0	0	0
Polemoniaceae	0	0	0
**Total**	**292**	**185**	**5462**
